# COVID-19 as a Vascular Disease: Lesson Learned from Imaging and Blood Biomarkers

**DOI:** 10.3390/diagnostics10070440

**Published:** 2020-06-29

**Authors:** Paolo Zamboni

**Affiliations:** Department of Surgery, Vascular Disease Centre University Hospital of Ferrara, 44124 Cona, Italy; paolozamboni@icloud.com; Tel.: +39-0532237694

**Keywords:** COVID-19, SARS-CoV-2, thrombosis, deep vein thrombosis, venous thromboembolism, ultrasound, vascular biomarkers, D-dimer, ischemia, lung ultrasound, contrast-enhanced ultrasound, pulmonary embolism

## Abstract

COVID-19, a disease initially thought to be prominently an interstitial pneumonia with varying degrees of severity, can be considered a vascular disease with regards to serious complications and causes of mortality. Quite recently, blood clots have emerged as the common factor unifying many of the symptoms initially attributed without an explanation to COVID-19. Cardiovascular biomarkers and particularly, D-dimer and troponin appear to be very powerful prognostic markers, signaling the need for earlier and more aggressive interventions and treatments in order to avoid and/or minimize arterial/venous thromboembolism and myocardial infarct. The ultrasound imaging patterns at both the lung and peripheral vascular level can also be very useful weapons that have the advantage of being able to monitor longitudinally the clinical picture, something that real-time PCR/nasopharyngeal swab is not able to do and that CT can only pursue with significant radiation exposure. A lesson learned in the early phase of the COVID-19 pandemic suggests quitting and starting again with targeted imaging and blood vascular biomarkers.

## 1. Critical Issues of the Current Diagnosis of SARS-CoV-2 Viral Infection

Italy was the first western country to be stricken by the coronavirus pandemic. From the outset, it became mandatory to identify and isolate COVID-19 patients, many of whom were then admitted to hospital. The most common symptoms of the disease at onset were fever, fatigue, dry cough, dyspnea, runny nose or other upper respiratory tract symptoms. Ageusia and anosmia were also found to be characteristic symptoms, albeit with more rare presentation, while gastrointestinal symptoms account for a minority of cases. Laboratory tests also found COVID-19 to be associated with changes in blood chemistry, with D-dimer, lactate dehydrogenase (LDH) and the aspartate transaminase to alanine transaminase ratio (AST–ALT) all showing interesting changes in the negative predictive value to positive predicted value (NPV–PPV) relationship [1,2].

As the COVID-19 outbreak progressed, chest X-rays were found to display relatively low sensitivity, whereas chest CT exhibited higher sensitivity scores, with the latter achieving: sensitivity of 97%, specificity of 25%, PPV of 65%, and NPV of 83%, with real-time PCR/nasopharyngeal swab (swab-PCR) as the reference method [2]. Given such a low specificity score, the use of dedicated software up to the introduction of artificial intelligence can allow chest CT to significantly improve the reporting speed and the diagnostic accuracy, as well [3,4]. An alternative approach that has great potential is lung ultrasound (LUS), which has been shown to be capable of diagnosing interstitial pneumonia with great accuracy compared with chest CT [5]. Indeed, the receiver operating characteristic curves for LUS have shown a strong relationship between sensitivity and specificity, with area under the curve scores of 0.95 and 0.93, respectively, achieved in two distinct meta-analyses [6,7].

Unfortunately, the use of swab-PCR as the primary diagnostic tool for COVID-19 leaves much to be desired. Indeed, of the 72,314 COVID-19 patients at the Wuhan University Hospital, only 62% had a positive swab-PCR [1], with diagnosis in the remaining 38% of cases achieved through contact history, symptoms, blood chemistry tests and pulmonary CT. Furthermore, in another large study, in 75% of patients with negative swab-PCR, chest CT demonstrated interstitial pneumonia often consistent with COVID-19 etiology. Moreover, by means of analysis of serial swab-PCR assays and CT scans, the mean interval time between the initial negative to positive swab-PCR result was 5.1 ± 1.5 days [2]. As such, there is general agreement that a negative swab-PCR test should be interpreted with caution and a repeat test may be needed [2,8,9].

Finally, we must not to forget serology testing for viral antibodies, which is an important indicator of previous exposure to the virus. In a longitudinal study, sensitivity and specificity for detecting seropositivity at ≥ 15 days following a positive SARS-CoV-2 swab-PCR result, was 100.0% and 98.7% when assaying for the panels of IgM and IgG. The median time to seropositivity observed for a reactive IgM and IgG result from the date of a positive PCR was 5 days (IQR: 2.75–9 days) and 4 days (IQR: 2.75–6.75 days), respectively [10].

## 2. The Endothelial Cell as a Target of SARS-CoV-2

The SARS-CoV-2 spike protein is known to bind to the angiotensin-converting enzyme 2 (ACE2) receptor on the surface of human cells. The spike protein is processed by membrane proteases, including TMPRSS2, and is internalized into the cell, leading to infection [11]. ACE2 receptors are abundantly expressed by endothelial cells. In a paper published in April 2020, it has been demonstrated that the SARS-CoV-2 virus can infect the endothelial cells in the lungs, heart, kidneys, liver, and intestines of patients with COVID-19 infection [11]. The demonstration of endothelial cell injury across vascular beds of different organs gives light to unexplained symptoms and clinical courses described in early reports of the COVID-19 pandemic. In particular, histological analysis revealed the presence of the virus within endothelial cells was associated with clusters of inflammatory cells. This finding suggests that SARS-CoV-2 infection initiates endothelial inflammation throughout the entire human organism, as well as apoptosis, something that explains the systemic macro and microcirculatory involvement in different vascular beds and their clinical sequelae in patients with COVID-19 [12]. Moreover, evidence of viral endothelial injury helps to explain why patients with pre-existing cardiovascular disease are particularly associated with adverse outcomes in COVID-19. Notwithstanding this, awareness that COVID-19 targets the endothelial cells provides a rationale for exploring several established cardiovascular therapies known to protect the endothelium in the hope of reducing viral replication [13,14,15,16,17].

## 3. Coagulation and Cardiovascular Biomarkers Predict COVID-19 Mortality

**Figure 1:**
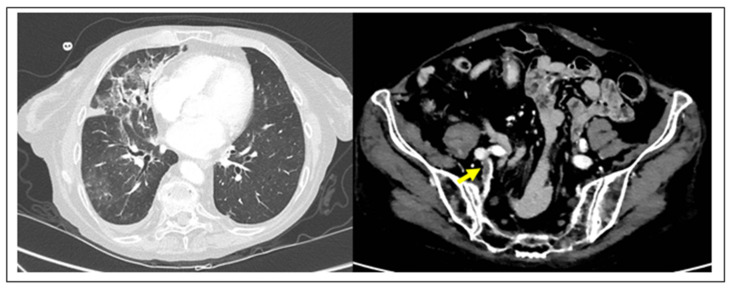
TC imaging of DVT complication in COVID-19 pneumonia. (**Left**) COVID-19 pneumonia with typical association of ground glass opacities and thickening of interlobular septa, particularly visible at the level of the right lung. (**Right**) The same case complicated by right hypogastric DVT (yellow arrow) (D-dimer 5.52 2.0 µg/mL).

In the past few months, blood clots have emerged as the common factor unifying many of the mysterious symptoms attributed to COVID-19, a disease that had initially been thought to largely affect the lungs in the form of pneumonia. Early findings in COVID-19 autopsies showed deep vein thrombosis (DVT) in 58% of cases, complicated by fatal venous thromboembolism (VTE) in 30% of patients. In addition, sudden cardiac death and kidney infarct complications were found in the other 30% of patients of this initial cohort [18]. Recently, the hypothesis that COVID-19 pneumonia might be complicated by VTE has been supported by an increased number of reports in the COVID-19 literature [19,20,21,22,23,24] (Figure 1).

Since the initial reports, an increase in circulating D-dimer levels has been reported, without being clarified if the cause was the cytokine storm of COVID-19 interstitial pneumonia or if there were overlapping thrombotic phenomena. Furthermore, increased levels of such a biomarker are associated with poor prognosis and/or death [19]. Indeed, it has been determined that a cutoff value 2.0 µg/mL of D-dimer can predict in-hospital mortality, with a sensitivity of 92.3% and a specificity of 83.3%. COVID-19 patients with D-dimer levels ≥2.0 µg/mL have been shown to have a significantly higher incidence of mortality when comparing to those who with D-dimer levels < 2.0 µg/mL (*p* < 0.001). Fatal VTE in course of COVID-19 is preceded by changes in blood coagulation biomarkers such as increased values of D-dimer, decreased antithrombin values, prothrombin time, and thrombin time [24]. The addition of systemic proinflammatory cytokines release as a consequence of endothelial inflammation, as well as the expression of the ACE2 receptors for SARS-CoV-2 on the membrane of the vascular muscle and endothelial cells, may help to explain why COVID-19 patients are also susceptible to arterial thrombosis, even in young non-arteriosclerotic individuals [25]. Furthermore, cerebral circulation may also be involved, as retrospective analysis in Wuhan revealed, where 6% of the deaths among COVID-19 patients were stroke-related [26].

Finally, elevated cardiac troponin levels are associated with myocardial injury, and in turn, with a fatal outcome in the clinical course of COVID-19 [27,28,29]. This is evident by the paradox that patients with underlying cardiovascular disease but without increased troponin achieve better outcomes than younger patients without comorbidities but higher troponin levels. In a single center study, the stratification of the mortality rate in the subgroups of patients during hospitalization for COVID-19 was respectively: 7.62% for patients without underlying chronic cardiovascular disease and normal troponin T levels; 13.33% for those with comorbidities and normal troponin levels; 37.50% for those without associated cardiovascular diseases but elevated troponin levels; 69.44% for those with both underlying cardiovascular diseases and elevated troponin. However, patients with underlying comorbidities were more likely to exhibit elevation of troponin T as compared with the patients without previous cardiovascular diseases, respectively 54.5% versus 13.2% [29]. Given this, it is important to triage patients with suspected COVID-19 according to their history of cardiovascular disease, assessing, at least, their D-dimer and troponin levels.

## 4. Vascular Therapeutic Implications

There is a growing body of evidence suggesting that SARS-CoV-2 can bind the glycosaminoglycans (GAGs), including heparin. The latter acts as a decoy, preferentially binding to the SARS-CoV-2 S1 spike protein and inhibiting SARS-CoV-2 entry into cells. Initial binding with heparin appears also to change the conformation of the spike protein inhibiting downstream binding and processing of the ACE2 receptor and TMPRSS2, respectively. It has been recently demonstrated that intact recombinant S1S2 spike protein from SARS-CoV-2 can bind to a human cell line that expresses ACE2 and TMPRSS2, and shown that unfractionated heparin and some low molecular weight heparins (LMWH), particularly enoxaparin in routine clinical use, determines a robust inhibition of S1S2 binding [30,31,32,33].

In light of what has been previously reported on the pathophysiological, diagnostic, and prognostic value of D-dimer, the dual role of heparin as a therapeutic weapon becomes clear—on the one hand, as a powerful inhibitor of the entry of the virus into cells, and on the other, as a preventer of the thromboembolic process. This is something which appears to be confirmed in clinical practice, since early analysis of in-hospital patients has revealed that anticoagulant treatment is associated with decreased mortality in COVID-19 patients. The 28-day mortality in COVID-19 patients with alteration of coagulation parameters including D-dimer in the LMWH group was significantly lower than in the non-user group. Again, the rate of mortality was significantly higher in patients with D-dimer >6-fold with respect to the upper limit of normality than in those below, respectively 52.4% versus 32.8% *p* = 0.017 [34].

## 5. Lung Ultrasound to Protect Admission to Hospitals and Surgical Services

**Figure 2:**
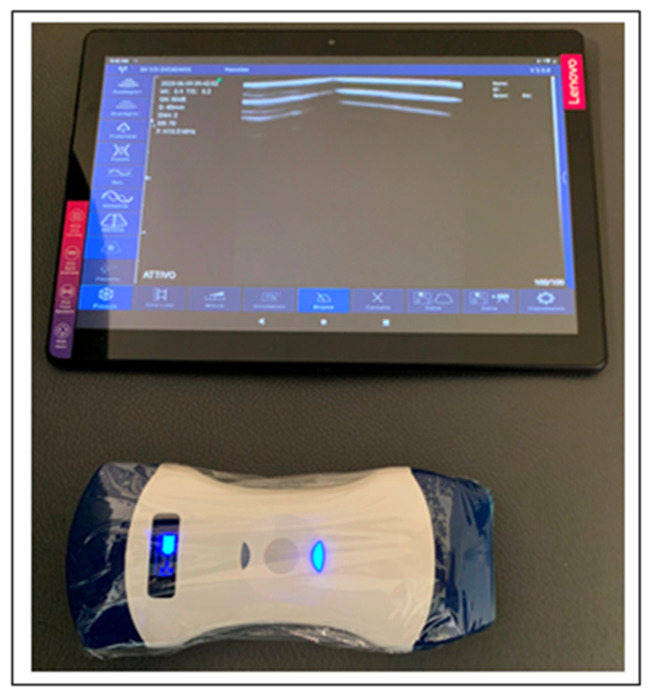
Wireless ultrasound equipment to avoid contamination. A wireless ultrasound probe with respectively a 4.5 mHz convex probe on the right side, and a 7.5 mHz linear probe on the left one. The transducer, wrapped with a single use plastic cover to avoid contamination, is wi-fi connected with the tablet which in turn, is encircled by a plastic film.

I do not want to comment here on the consequences of the high percentage of false negatives of swab-PCR in a pandemic, nor discuss the reasons for swab-PCR vulnerability. Rather, we simply point out that there is an urgent need for a faster and more sensitive test to regulate access to hospitals, including the surgical unit, especially in emergency circumstances. As such, this issue continues to be an open diagnostic problem that affects both patients and health care professionals. Looking at the current diagnosis of COVID-19, it would appear that both chest CT and LUS have a valuable role to play in the triaging of patients into hospitals, especially in cases of emergency surgery or in situations where surgery cannot be procrastinated. However, the use of CT scans to identify COVID-19 carriers has the disadvantage that it increases their exposure to radiation, as well as being a relatively costly health care procedure [35]. Recently, a group of LUS experts developed a standardized protocol for investigation of COVID-19 pneumonia [36]. To this end, they suggested to use a tablet E-connected to a wireless probe, with both wrapped in single use plastic film covers [36,37,38,39,40]. As such, this strategy minimizes the risk of contamination and facilitates easy disinfection and sterilization of equipment (Figure 2).

**Figure 3:**
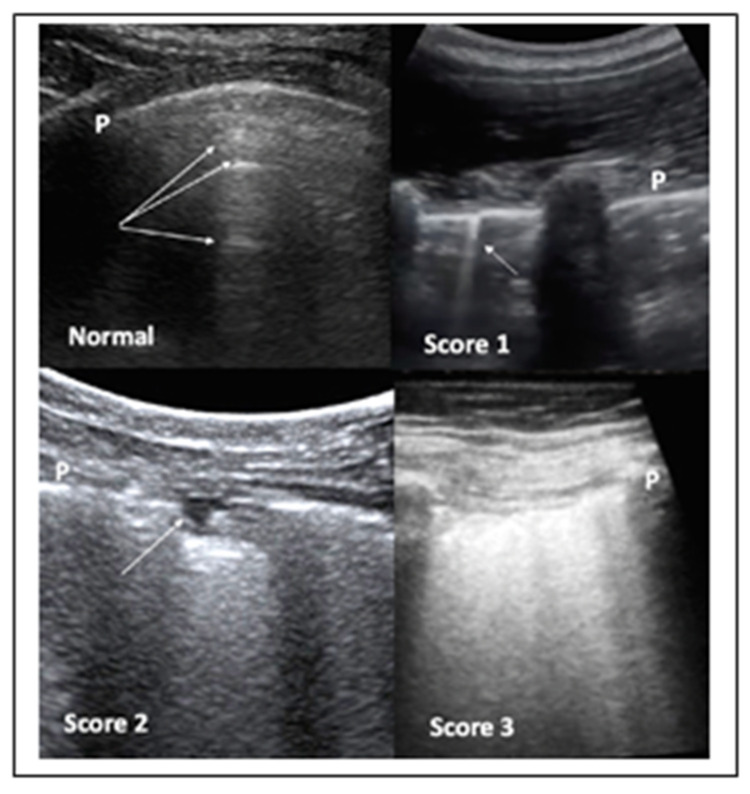
LUS aspects of COVID-19 pneumonia. Top left panel—LUS in normal cases. The pleura line (P) is sliding, mobile at breathing, and hyperechoic. The arrows indicate the A lines, which are horizontal, not mobile lines below the pleura line. Top right panel—LUS in COVID-19 patient: the presence of irregularities of the pleura line (P) coupled with vertical, comet-like, mobile B lines (arrow) become apparent. These findings are frequently bilateral and alternate with areas of normality, as shown in this panel. Bottom left panel—LUS in COVID-19 patient: the pleura line P is broken. Subpleural triangular dark area (arrow) with a hyperechoic floor is defined a subpleural consolidation area. Bottom right panel—LUS in COVID-19 patient: the so-called “white lung”.

It has been also suggested that LUS should involve two operators in the acquisition protocol in order to reduce their exposure time to COVID-19 patients, with the first scanning and the second one storing the images. Both convex and linear probes can be used. Moreover, the standard LUS investigation is composed by 14 intercostal windows, seven for each side: three posteriorly along the paravertebral line; two laterally along the mid-axillary line; two anteriorly along the mid-clavicular line, these latter ones below and above the inter-nipple line, respectively. The LUS in patients not able to maintain a sitting position can be performed in lateral decubitus. COVID-19 pneumonia can be also scored for severity by LUS (Figure 3).

The introduction of the severity score leads us to prefer LUS to both swab-PCR and CT scan for the following clinical needs:

- When following-up the evolution of COVID-19 pneumonia, in situations where the use of a CT scan would expose the patient to an excess of radiation.
- When monitoring longitudinally health care professionals. Since it is expected that the pandemic will continue for some time, it will also be necessary to monitor medical staff. For this purpose, LUS would be ideal. Based on the incubation time, it would seem reasonable to repeat the survey every two weeks.

Ideally, preliminary LUS screening would be undertaken before admission, in general, to hospital, and particularly to surgical departments. In the case of a positive LUS test, ideally corroborated by hepatic and coagulation blood markers (as discussed above), the patient would be isolated and surgically treated according to COVID-19 hospital protocol. By contrast, in the case of negative LUS outcome and blood laboratory test, the patient would follow the standard route. In a department of surgery, this approach could represent a fast, sensitive, cost effective assessment, which would protect other patients and health care staff during the pandemic. Finally, LUS would permit the avoidance of X-rays and can be rapidly performed by the surgeons, reducing the overwhelming of the radiology services. From this point of view, the development of an e-learning LUS educational program represents a matter of urgency.

## 6. Ultrasound in VTE

As described above, an elevation in D-dimer levels is a common finding in patients with COVID-19. Throughout the pandemic, several reported cases have associated this biomarker with acute DVT and/or VTE. Clinical suspicion of VTE is thought to be higher in cases with DVT symptoms, with rapid and disproportionate hypoxemia, or acute unexplained right ventricular dysfunction [12]. At the beginning of the pandemic, the presence of elevated D-dimer did not warrant routine ultrasound investigation. Ultrasound investigation, currently used to diagnose DVT, was therefore not adopted, given the risk of transmitting infection to other patients or health care workers. However, with the subsequent introduction of wireless ultrasound probes, which can be rapidly covered in single use transparent films, has completely changed this scenario (Figure 2). As such, LUS is rapidly becoming a useful, cost effective, and safe diagnostic tool for identifying and clinically assessing COVID-19 pneumonia.

**Figure 4:**
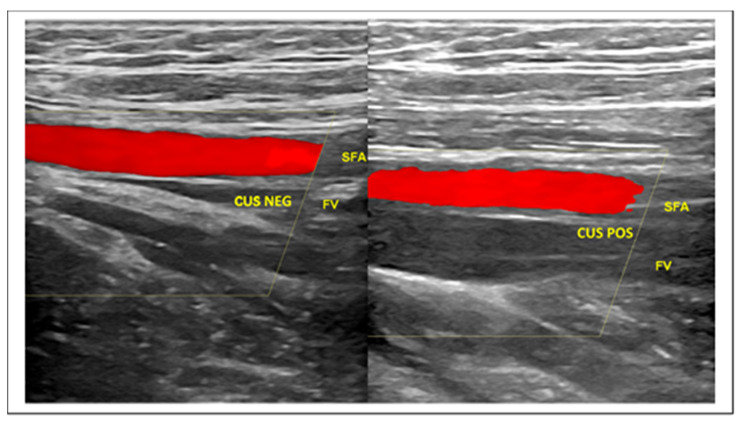
CUS maneuver for rapid identification of DVT. (**Left**) The real time compression by the ultrasound probe of the femoral vessels at the thigh determines the collapse of the femoral vein. CUS maneuver negative for DVT. (**Right**) The same maneuver in the case of femoral vein thrombosis does not demonstrate venous collapse. CUS maneuver positive for DVT. Legend: SFA—Superficial Femoral Artery; CUS NEG—Negative for thrombosis Compression Ultrasonography; FV—Femoral Vein; CUS POS—Positive for thrombosis Compression Ultrasonography.

The first-line imaging test in the diagnostic management of patients presenting with clinically suspected DVT is compression ultrasonography (CUS), a powerful ultrasound biomarker [41,42,43] (Figure 4).

The ultrasound scan might include a second CUS examination after 5 to 10 days following an initial negative CUS to evaluate if a possible distal DVT has propagated to the proximal veins. Single limited, serial limited, and whole-leg CUS are the current imaging strategies for the diagnosis of DVT. Preference for one strategy over the other differs between centers and sonographers [41,42,43,44].

**Figure 5:**
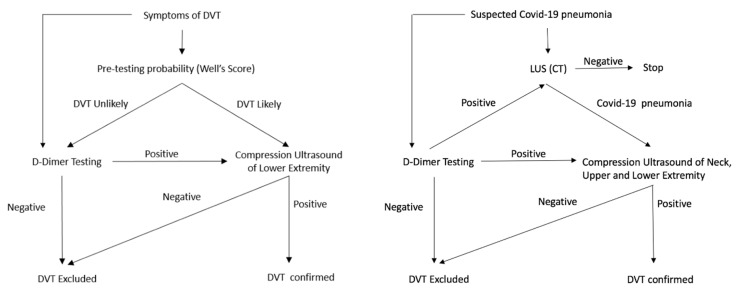
Current diagnostic flowchart for suspected DVT as compared to diagnostic flowchart for suspected COVID-19. (**Right**) current diagnostic flowchart used in case of suspected DVT. (**Left**) in the COVID-19 era, the flowchart for suspected patients would include both D-dimer and LUS. Positive cases should be immediately investigated for DVT by CUS at the level of the neck, upper, and lower extremities, respectively.

Due to frequent association with VTE, it could be reasonable to complete the LUS screening protocol with CUS investigation at the level of the jugular, subclavian, femoral, popliteal, and calf muscular venous segment, according to the above protocols. The addition of ultrasound venous images to the LUS protocol takes just a few minutes and is able to provide fundamental prognostic and therapeutic information (Figure 4). Finally, taking into account all the above data for the diagnosis of COVID-19 and the frequent VTE complications, the usual DVT diagnostic algorithm could be usefully modified by means of blood and ultrasound vascular biomarkers, according to a novel flowchart illustrated in Figure 5.

## 7. Contrast-Enhanced Ultrasound and Pulmonary Embolism

As mentioned above, both CT and LUS detect subpleural consolidation areas in the course of COVID-19 pneumonia. Some authors, in the light of frequent VTE complications, have raised the question as to whether these areas of consolidation might actually be indicative of segmental pulmonary embolus [45]. To answer their question, it has recently been proposed that contrast-enhanced ultrasound (CEUS) be used when performing LUS scans [46]. Tee et al., were able to demonstrate by means of CEUS that irregular areas of subpleural consolidation at LUS are avascular and therefore, most likely represent microinfarcts. Conversely, consolidation of non-thrombotic origin would be seen to have some enhancement at CEUS investigation. The same cannot be seen by using CT due to the superior spatial resolution of ultrasound.

## 8. Final Remarks

Vascular biomarkers confirm that COVID-19, a disease initially thought to be exclusively an interstitial pneumonia with varying degrees of severity, can also be considered a vascular disease, especially with regards to more serious complications and causes of mortality. Particularly, both D-dimer and troponin appear to be very powerful prognostic markers, signaling the need for earlier and more aggressive interventions and treatments. The ultrasound imaging pattern at both lung and peripheral vascular level can also be very useful weapons that have the advantage of being able to monitor longitudinally the clinical picture, something that swab-PCR is not able to do and that CT can only pursue with significant radiation exposure.
